# Altered Metabolomics in Bipolar Depression With Gastrointestinal Symptoms

**DOI:** 10.3389/fpsyt.2022.861285

**Published:** 2022-05-24

**Authors:** Xiang-Jie Guo, Yan-Bing Xiong, Yuan Jia, Xiao-Hong Cui, Wen-Ze Wu, Jun-Sheng Tian, Hong Yang, Yan Ren

**Affiliations:** ^1^Department of Forensic Medicine, Shanxi Medical University, Taiyuan, China; ^2^Department of Psychiatry, Shanxi Bethune Hospital, Shanxi Academy of Medical Sciences, Tongji Shanxi Hospital, Third Hospital of Shanxi Medical University, Taiyuan, China; ^3^Tongji Hospital, Tongji Medical College, Huazhong University of Science and Technology, Wuhan, China; ^4^Modern Research Center for Traditional Chinese Medicine, Shanxi University, Taiyuan, China; ^5^The Key Laboratory of Chemical Biology and Molecular Engineering of Ministry of Education, Shanxi University, Taiyuan, China; ^6^Shanxi Provincial Key Laboratory of Brain Science and Neuropsychiatric Diseases, Taiyuan, China

**Keywords:** bipolar disorder, gastrointestinal symptoms, metabonomics, biomarker, gut microbiome

## Abstract

**Objective:**

Although gastrointestinal (GI) symptoms are very common in patients with bipolar disorder (BD), Few studies have researched the pathomechanism behind these symptoms. In the present study, we aim at elucidate the pathomechanism of GI symptoms in BD through metabolomic analysis.

**Method:**

BD patients were recruited from Shanxi Bethune Hospital that divided into two groups, each group assessed with the 24-item Hamilton Depression Rating Scale (HAMD-24) according to the presence or absence of GI symptoms. Healthy controls were recruited from the medical examination center of the same hospital. Differential metabolites were identified and further analyzed using Metabo Analyst 3.0 to identify associated metabolic pathways.

**Results:**

There were significantly higher HAMD-24 scores in the GI symptoms group than that of non-GI symptoms group (*p* = 0.007). Based on metabolomic analysis results, we found that the common disturbances metabolic pathway of both two patients groups was ketone body metabolism, and the unique disturbances metabolic pathways of BD with GI symptoms were fatty acid biosynthesis and tyrosine metabolism, and these changes were independent of dietary habits.

**Conclusion:**

BD patients with GI symptoms exhibited disturbances in fatty acid and tyrosine metabolism, perhaps suggesting that the GI symptoms in BD patients are related to disturbances of the gut microbiome. Both groups of patients jointly exhibit disturbances of ketone body metabolism, which may serve as a biomarker for the pathogenesis of BD patients.

## Introduction

Bipolar disorder (BD) is a chronic psychiatric disorder characterized by recurrent episodes of depression (bipolar depression), mania (bipolar mania), and hypomania, which affecting 1–3% of the population worldwide (1, 2). On the bipolar spectrum, bipolar depression is the leading cause of morbidity in patients with bipolar disorder, at least 50% of patients initially present with a depressive episode (3). Mood disorders or emotional symptoms, such as depression, are common among those who seek help for functional somatic symptoms. According to the studies, ~69% of patients with major depressive disorder (MDD) experience somatic symptoms (4, 5) and over 45% of MDD seek medical attention mainly for these (6). According to a meta-analysis, the estimated prevalence of somatic symptoms in BD was 47.8%, a rate nearly double that of the healthy controls, and a rate similar to MDD (7). Studies have likewise suggested that somatic symptoms of BD may be an independent risk factor of disease severity, suicidal ideation, and rapid-cycling disease processes, implying that somatic and psychological symptoms must be co-managed in severe mental illness (8). Therefore, somatic symptoms may be key indicators of the severity and prognosis of BD.

Gastrointestinal (GI) symptoms as the most commonly reported group of somatic symptoms in affective disorders, which have a strong relationship with anxiety and depressive disorders (9). The majority of patients presenting with major depressive and anxiety also complain of GI symptoms, such as nausea, bloating, decreased appetite, etc. One study based on the general population found that 54% of those with depressive symptoms had complaints of GI discomfort, which was much higher than 29% of controls without affective disorders. In addition, the GI symptoms group had more severe affective symptoms as well as a decreased quality of life than the non-GI symptoms group (10). Another study found that there is a strong association between symptoms of affectivity and GI symptoms in BD, the findings are consistent with those reported in MDD (11).

Studying the causal connection between the onset of GI symptoms and affective symptoms is difficult because the onset of both symptoms is insidious and the course fluctuates widely (12). Previous studies have shown that there may be a common pathophysiological mechanism between depressive and GI symptoms, which may refer to the neuroendocrine system, neural plasticity, inflammatory response cascade and gut microbiome (13). Metabolomics, a branch of systems biology that has been widely used in many fields, can help us understand the mechanisms by which the gut microbiota could drive symptom. With the development of effective analytical techniques and methods, the discovery of specific biomarkers through the analysis of fluids and tissues has made a significant contribution to the understanding of the basis of disease (14). Four main matrixes (i.e. feces, urine, plasma and serum) have been used to analyzing the metabolites involved in the gut-brain axis by metabolomics, when focusing on plasma samples, the main goal is to remove the proteins from the sample prior to metabolomics analysis (15). We can maximize the metabolomics information of the samples by proton nuclear magnetic resonance (^1^H NMR), gas chromatography-mass spectrometer (GC-MS), liquid chromatography-mass spectrometry (LC-MS) combined with multivariate data analysis.

GI pathologies have long been known as a common comorbidity of BD and other psychological disorders, further confirming the theory that GI pathology and psychological disorders are interrelated. A meta-analysis consisting of 177,117 irritable bowel syndrome (IBS) patients and 192,092 healthy controls showed a significant increase in the prevalence of BD in patients with IBS compared to healthy participants (16). In IBS patients, ^1^H NMR spectroscopy showed the short-chain fatty acids (SCFAs) propionate, butyrate, and acetate to be significantly lower in the stool samples of IBS compared to healthy controls (17).

Thus, abnormal metabolic may be associated with the onset and development of GI symptoms. However, few studies have investigated the changes in metabolic in BD patients with GI symptoms. In the present study, we sought to elucidate the biochemical basis of GI symptoms by comparing metabonomics between BD patients with and without GI symptoms. We further sought to determine if these metabolic pathways were risk factors for GI symptoms in BD patients.

## Materials and Methods

### Participants

Fifty-nine BD patients were recruited from Shanxi Bethune Hospital. All subjects and their guardians voluntarily participated in the study and gave informed consent. Experienced and licensed psychiatrists participated in the recruiting procedure. For inclusion in this study, the diagnoses were validated with the DSM-5 (Diagnostic and Statistical Manual of Mental Disorders, Fifth Edition). The exclusion criteria for this study included any physical or other mental disorders and drug abuse. All patients have never been treated with medication or have not been treated in the last month. A control group of 10 healthy controls were recruited by advertisement and had no DSM-IV axis I disorders. The Ethics Committee of Shanxi Bethune Hospital reviewed and approved this study and all subjects provided informed consent prior to inclusion.

Eligible patients with BD were divided into two groups based on various GI symptoms scores from the HAMD-24 (item 12) and Young Mania Rating Scale (YMRS). The scores of YMRS for all enrolled patients were <5. The severity of GI symptoms of HAMD-24 is divided into three levels from 0 to 2. Zero means no GI symptoms, 1 means the most severe GI symptoms leading to mild occasional discomfort, and 2 means the most severe symptoms leading to frequent discomfort. Among the eligible patients with BD, 34 patients reported at least one GI symptom (GI symptoms group), and 25 patients reported no GI symptoms (non-GI symptoms group). Furthermore, all of the healthy controls were free of any GI symptoms.

### Sample Collection

The blood samples were collected from all subjects in the morning after 12 h of fasting by the professional staff of Shanxi Bethune Hospital. After clotting, the blood was mixed upside down and stored at room temperature for half an hour. Then centrifuged at 3,000 r/min for 15 min, and the supernatant (plasma) was taken by pipette and uniformly transferred to clean eppendorf tubes, and stored at −80°C prior to analysis.

### NMR Acquisition

The samples stored at −80°C were thawed at 0°C and centrifuge at 3,000 rpm for 15 min. Four hundred and fifty μL of plasma was mixed with 900 μL of methanol and centrifuged at 13,000 rpm for 20 min at 4°C to pellet the proteins, then 1,000 μL of supernatant was transferred to a clean vial. Another 900 μL of methanol was added and the proteins were removed by centrifugation at 4°C for 20 min at 13,000 rpm. Finally, 1,800 μL of the supernatant was dried in a nitrogen stream, and 600 μL of dry-mixed samples [phosphate buffer (0.2 M Na_2_HPO_4_/NaH_2_PO_4_, pH = 7.4)] containing sodium 3-trimethylsilyl D_2_O-(2, 2, 3, 3-d4) 1-propionate (TSP, 0.01%) to reduce chemical shift changes and remove any precipitates by centrifugation (13,000 rpm, 10 min at 4°C) at that time. Five hundred and fifty μL of supernatant was transferred into a 5-mm NMR tubes for NMR analysis (18).

The ^1^H NMR spectra of the plasma samples were obtained using a Bruker 600 MHz AVANCE III NMR spectrometer (Bruker Biospin, Rheinstetten, Germany) operating at a ^1^H frequency of 600.13 MHz and a temperature of 298 K. The broad signals with short lateral relaxation times of proteins and lipoproteins were attenuated using one-dimensional Carr-Purcell-Merboom-Gill (CPMG, RD-90-(τcp-180-τcp)-capture) and spin-spin relaxation delay of 320 ms. The ^1^H NMR spectrum of each sample consisted of 64 scans with an acquisition time of 5 min and parameters of spectral width of 12019.2 Hz, spectral width of 65,536 points, and pulse width (PW) = 30 (12.7 ms), and relaxation delay (RD) = 1.0 s (18).

### Data Processing and Statistical Analyses

All of the acquired ^1^H NMR spectra were manually phased, and the baseline was set using MestReNova software (Mestrelab Research, Santiago de Compostella, Spain). The chemical shift of creatinine (δ3.04, -CH3) was used as a calibration standard. The region in the range of chemical shift values δ 4.7 ~ 5.2 ppm was artificially removed to eliminate the water peak. All NMR spectra were superimposed and the δ 0.8–9.0 ppm in the spectra were integrated into the base unit of δ 0.01 ppm (19).

The processed ^1^H NMR spectrum profile data were introduced into SIMCA-P 14.1 (Umetrics, Sweden) for multivariate analysis. Partial leastsquare-discrimination analysis (PLS-DA) and orthogonal projection to latent structure-discriminate analysis (OPLS-DA) were employed. The quality of the PLS-DA model was described by the parameters for model fitness (R^2^) and predictive ability (Q^2^), where a large R^2^ (close to 1) and Q^2^ (Q^2^ ≥ 0.5) indicate a good model. Next, the PLS-DA model was validated by the response values of the permutation test in which the class membership was randomly shuffled 200 times. The result indicated a lack of over-fitting when the new R^2^ and Q^2^ values were lower than the original ones (20). In the OPLS-DA model, the *p*-values of cross-validated analysis of variance (CV-ANOVA) (*p* < 0.05) was used to indicate the significance level of population separation. To further understand the underlying variables that contribute to differentiation, we performed an S-plot analysis of the OPLS-DA model, where each coordinate reflects a NMR region used to define metabolites that contribute significantly to group separation. The key metabolites needed to differentiate groups were selected from the results of the variable importance (VIP) of the items analyzed by the established OPLS-DA model (18).

To determine the pathways involved in the metabolites where the differences occurred, they were further introduced into Metabo Analyst 3.0 (http://www.metaboanalyst.ca/) to perform Pathway analysis using the human Pathway library. Pathways were screened based on the *p* value of pathway enrichment and the impact value of pathway topology analysis.

Data analyses were performed using SPSS 20.0 (IBM, Chicago, IL, United States). A *t*-test was used to evaluate the significant differences in the selected signals of the main metabolites that were responsible for class discrimination. All clinical scale data were expressed as the mean ± SD. Continuous variables were checked using one-way analysis of variance (ANOVA) and classified variables using the Chi-square test. A *p* value of 0.05 or below was considered significant for demographic analysis.

## Results

### Demographic Data Comparisons

As shown in Table 1, there were no significant differences in age (*F* = 0.109, *p* = 0.897), gender (**χ2** = 4.261, *p* = 0.119), or body mass index (*F* = 0.501, *p* = 0.608) among the three groups. There were no significant differences in age of disease onset (*F* = 0.141, *p* = 0.709), duration of illness (*F* = 0.159, *p* = 0.691) between two patients group. However, the total HAMD-24 scores in the GI symptoms group were greater than that of the non-GI symptoms group (*F* = 7.761, *p* = 0.007). Furthermore, there were no significant differences in dietary habits among the three groups, include how often drink sugary drinks or eat desserts (χ2 = 6.510, *p* = 0.164), what kind of meat do eat (χ2 = 5.964, *p* = 0.202), and what kind of oil do use for cooking (χ2 = 1.808, *p* = 0.771).

**Table 1:** Demographic and clinical characteristics of all participants.

**Variable**		**Group**			**Analysis**		
		**GI (*N* = 25)**	**Non-GI (*N* = 34)**	**HC (*n* = 10)**	**df**	**F/χ2**	***p***
Age (year)		27.16 ± 9.02	28.29 ± 12.08	28.50 ± 3.10	66	0.109	0.897
BMI		22.16 ± 2.97	22.65 ± 4.35	21.30 ± 3.68	66	0.501	0.608
Onset age (year)		21.40 ± 8.15	22.35 ± 10.58	-	57	0.141	0.709
Duration of illness (months)		37.32 ± 43.30	41.82 ± 42.52	-	57	0.159	0.691
The total scores of HAMD-24		31.68 ± 5.57	27.03 ± 6.84	-	57	7.761	0.007
Gender	Male	13	9	3	2	4.261	0.119
	Female	12	25	7			
Drink sugary drinks or eat desserts	<3 times /week	16	18	6	2	6.510	0.164
	3–6 times /week	6	6	4			
	>6 times /week	3	10	0			
kind of meat do eat	Don't eat meat	3	0	1	2	5.964	0.202
	Main lean meat	18	24	8			
	Each half	4	10	1			
	Main fat	0	0	0			
Cooking oil	Total vegetable oil	8	10	3	2	1.808	0.771
	Main vegetable oil	12	16	3			
	Each half	5	8	4			
	Main animal oil	0	0	0			

### ^1^H NMR Spectroscopy Data Analysis

**Figure 1:**
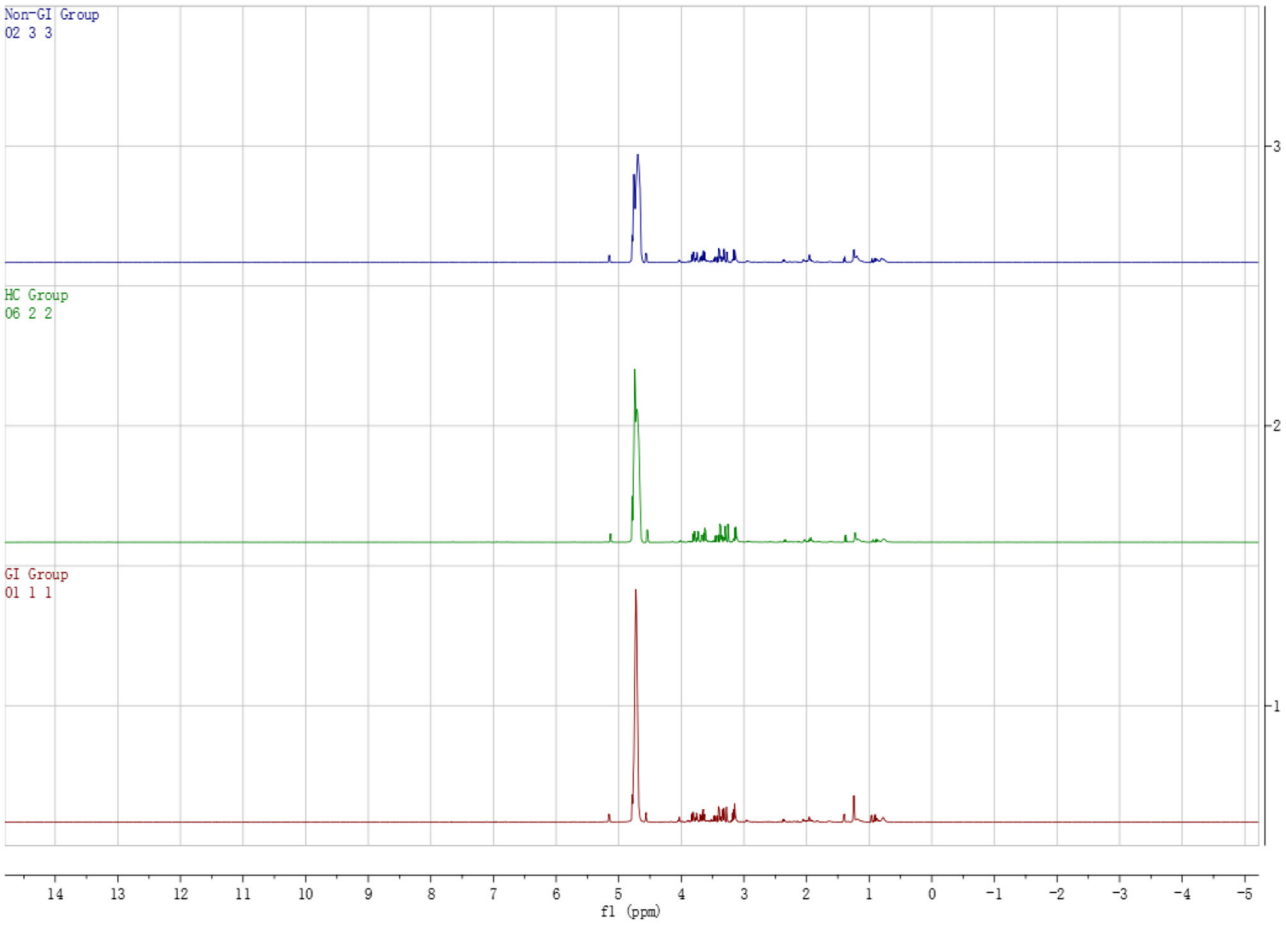
Typical ^1^H NMR spectrum of plasma in BD patients groups and healthy controls group.

Figure 1 shows the typical ^1^H NMR spectrum profiles of BD with GI symptoms, BD without GI symptoms and HC groups. By looking up the human metabolome database (HMDB) (http://www.hmdb.ca/) and related articles over the years, we identified 29 small molecule compounds in the ^1^H NMR spectrum profiles (Table 2).

**Table 2:** Peak attribution in ^1^H-NMR spectra of differential metabolites.

**No**.	**Metabolites**	**Chemical shift**
1	Lipid	0.874 (m)
2	Pantothenate	0.907 (s)
3	Isoleucine	0.949 (t)
4	Leucine	0.961 (t)
5	3-hydroxybutyric acid	1.21 (d)
6	Lactate	1.33 (d)
7	Acetate	1.927 (s)
8	O-acetyl glycoproteins	2.14 (s)
9	Acetoacetate	2.28 (s), 3.44 (s)
10	Methionine	2.14 (s)
11	Guanidinoacetate	3.80 (s)
12	Uracil	5.81 (d, 7.7 Hz), 7.55 (d, 7.7 Hz)
13	Histidine	7.04 (s), 7.84 (s)
14	Dimethylglycine	2.92 (s), 3.70 (s)
15	Creatine	3.04 (s), 3.93 (s)
16	Acetylcholine	3.23 (s)
17	Taurine	3.27 (t, J = 6.6 Hz), 3.42 (t, J = 6.6 Hz)
18	Scyllo-inositol	3.36 (s)
19	3-D-hydroxybutyrate	1.20 (d)
20	Betaine	3.27 (m)
21	Glyceryl	3.67 (m), 3.78 (m)
22	Citrulline	3.73 (s)
23	N-acetyl-glycoproteins	2.05 (s)
24	Glutamate	2.06 (m), 2.14 (m), 2.36 (m)
25	Glutamine	2.14 (m)
26	Acetone	2.23 (s)
27	Acetoacetate	2.28 (s), 3.44 (s)
28	Citrate	2.53 (d, 16.1 Hz), 2.70 (d, 16.1 Hz)
29	Choline	3.20 (s), 4.06 (m)

**Figure 2:**
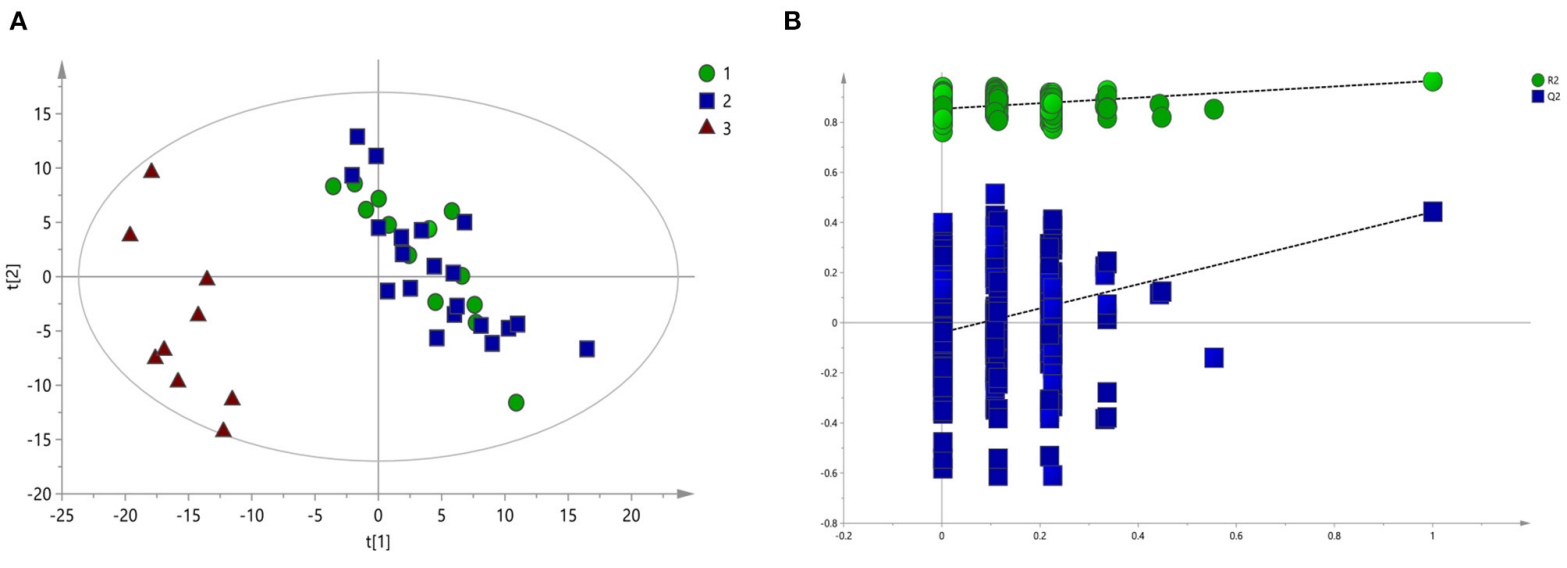
PLS-DA of ^1^H NMR spectra of urine samples from BD patients and HC groups. **(A)** PLS-DA score plots of ^1^H NMR spectra in which GI group (circle), Non-GI group (square) were obviously separated from HC group (triangle). **(B)** 200-iteration permutation test map of the PLS-DA model.

To screen the potential differential metabolites of the three groups, the ^1^H-NMR spectral data obtained were first subjected to PLS-DA. The score plot of the PLS-DA model showed that BD patients (whether or not with gastrointestinal symptoms) and HC could be clearly separated with little overlap (Figure 2A). In order to further verify the PLS-DA model, 200 iteration permutation tests were conducted (Figure 2B). The corresponding permuted values (bottom left) were all lower than the original R^2^ and Q^2^ values (top right), which meant the PLS-DA model was not over-fitted.

According to serum macro profile analysis of each group, it could be seen that HC was significantly separated from BD patients, indicating that endogenous metabolites in the BD patients group were changed compared with the HC group. However, There was no significant difference between the BD patients group with gastrointestinal symptoms and BD patients group without gastrointestinal symptoms. The above results could not observe the specific regulation of endogenous metabolites. Therefore, pairwise analysis in each group were required to find the differential metabolites.

### Plasma Metabolite and Metabolic Pathways Differences Between the GI Group and HC Group

**Figure 3:**
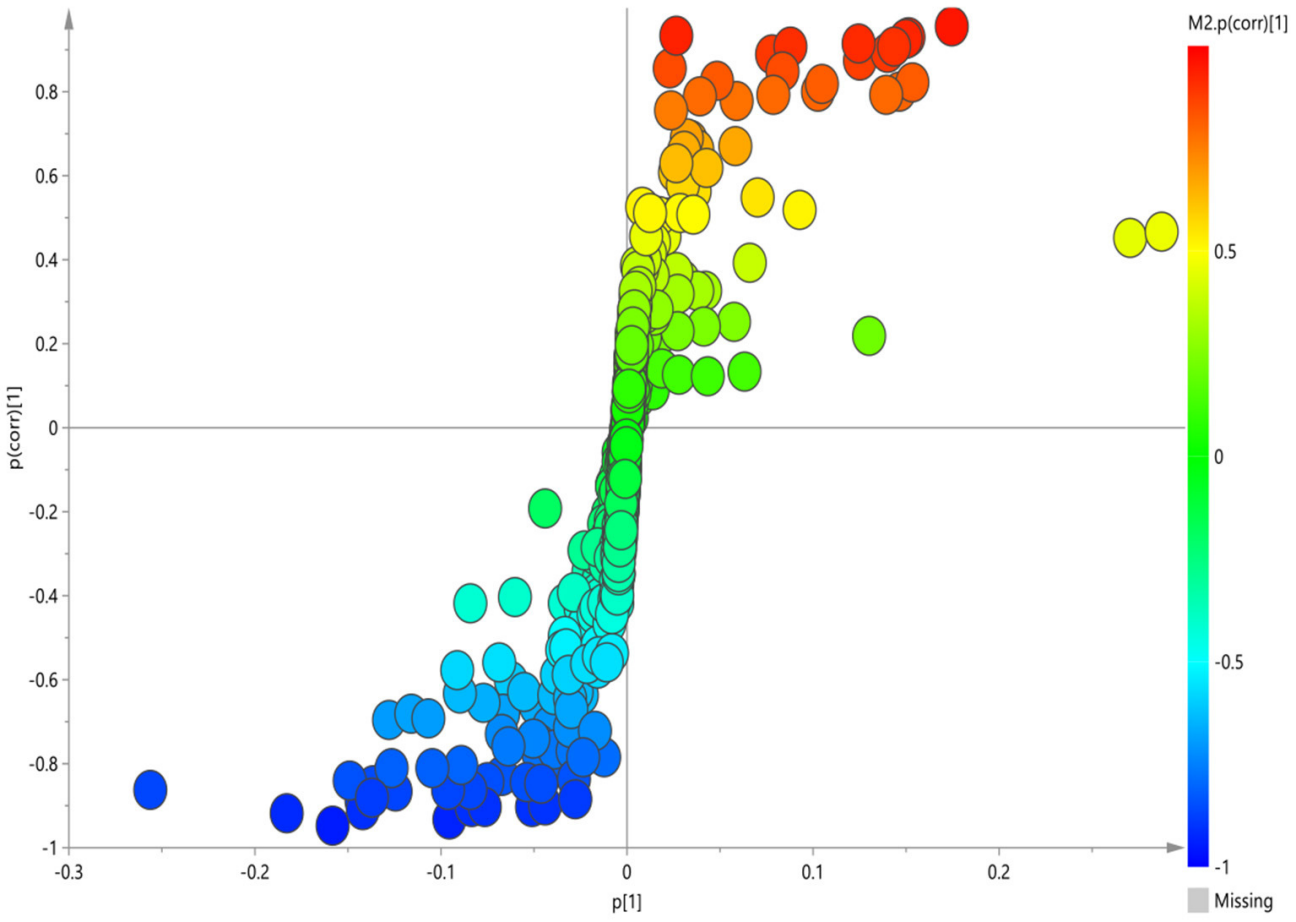
S-plot of ^1^H-NMR spectra between HC and GI group.

The corresponding OPLS-DA loading plot was further established between GI group and HC group to improve the classification of the different groups, as well as for biomarker screening, according to the S-plot (Figure 3), 10 key metabolites were especially meaningful for the distinction between two groups (VIP > 1 and *p* < 0.05; Table 3). Compared to HC, BD with GI symptoms were characterized by significantly higher levels of lipid, pantothenate, N-acetyl-glycoprotein, ascorbic acid, and significantly lower levels of 3-D-hydroxybutyric acid, acetate, acetoacetate, glyceryl and β- glucose.

**Table 3:** The peak area of metabolites in serum ^1^H-NMR spectra of HC group and GI group.

**Metabolites**	**Peak area after normalization**	
	**HC**	**GI**
Lipid	0.314 ± 0.079	0.467 ± 0.116
Pantothenate	0.245 ± 0.053	0.401 ± 0.098
3-D-hydroxybutyric acid	0.532 ± 0.192	0.127 ± 0.094
Acetate	0.717 ± 0.105	0.400 ± 0.131
N-acetyl-glycoproteins	0.120 ± 0.066	0.378 ± 0.082
Acetoacetate	0.623 ± 0.117	0.317 ± 0.132
Glyceryl	1.161 ± 0.192	0.563 ± 0.305
Ascorbic acid	0.231 ± 0.389	0.548 ± 0.116
β- glucose	10.432 ± 3.295	6.841 ± 4.178

**Figure 4:**
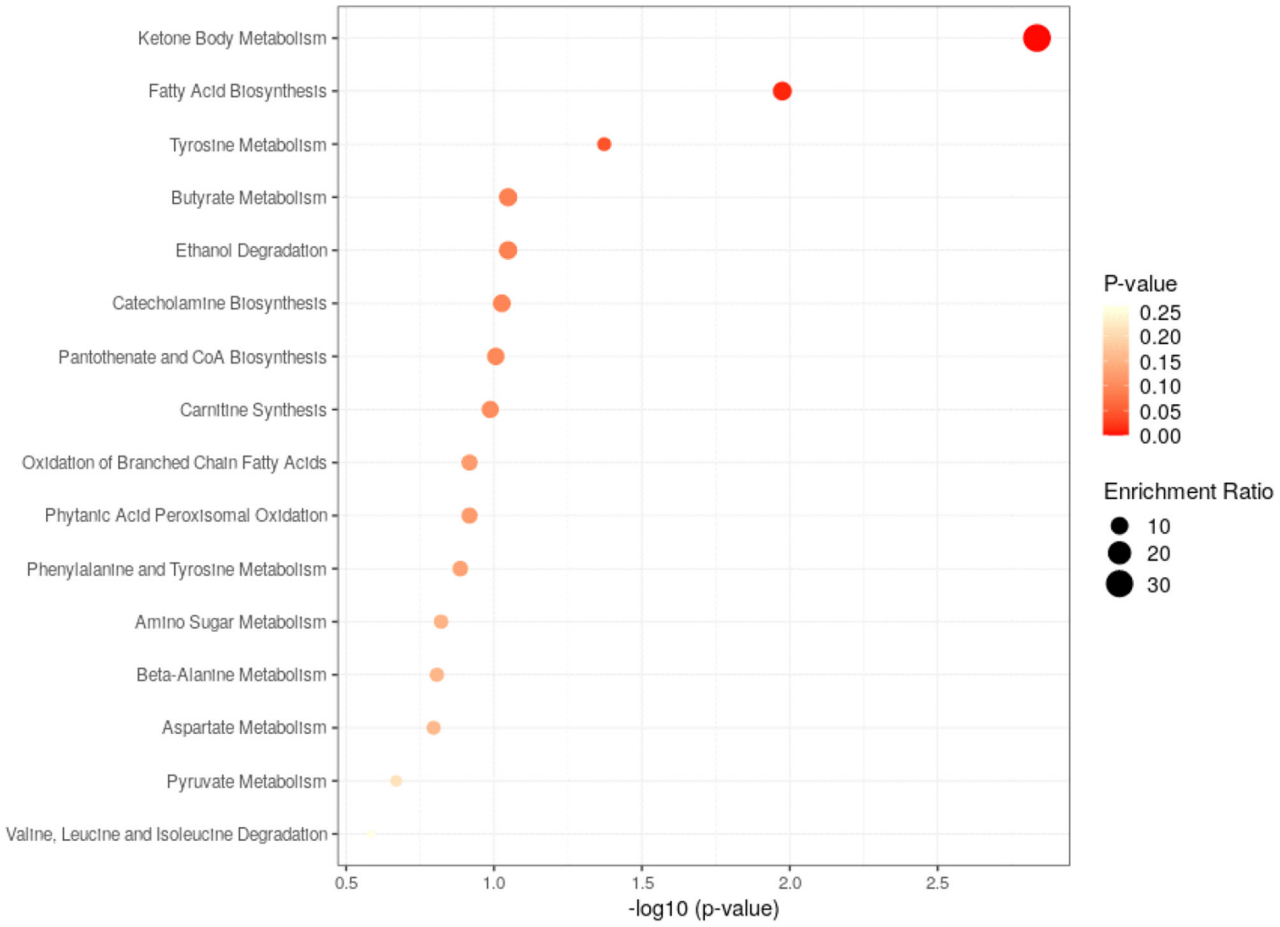
Metabolic pathways analysis of the differential metabolites found between HC and GI group.

Pathway analysis of differential metabolites was performed to reveal biological implications. Differential metabolites were analyzed using the Metabo Analyst 3.0 (http://www.metaboanalyst.ca/) platform, and the main involved metabolic pathways were identified. Among these, ketone body metabolism, fatty acid biosynthesis and tyrosine metabolism were more closely related to BD patients with GI symptoms. The analysis results are shown in Figure 4.

### Plasma Metabolite and Metabolic Pathways Differences Between the Non-GI Group and HC Group

**Figure 5:**
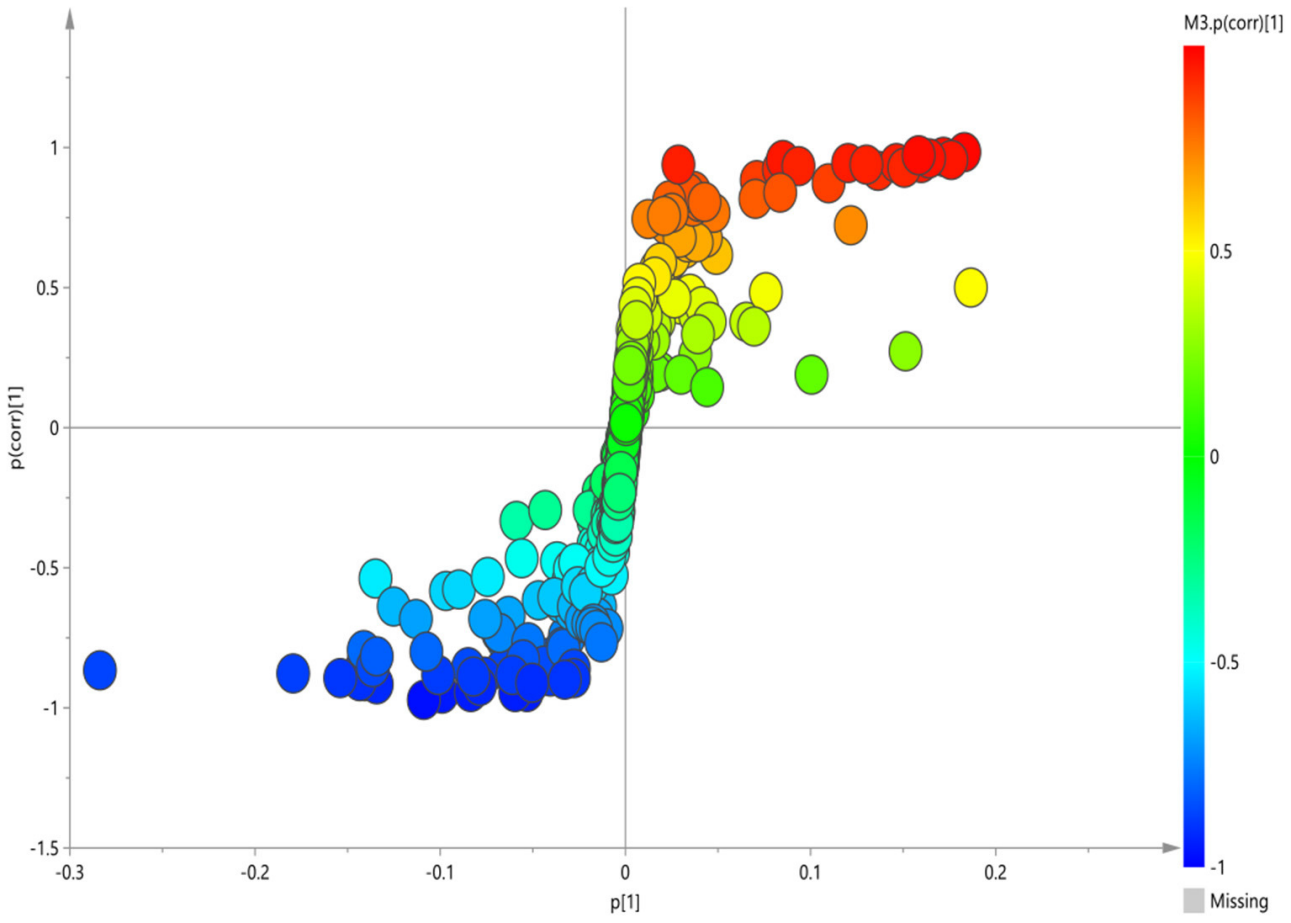
S-plot of ^1^H-NMR spectra between HC and non-GI group.

The corresponding S-plot was further established between Non-GI group and HC group (Figure 5). According to the S-plot, 10 key metabolites were especially meaningful for the distinction between two groups (VIP > 1 and *p* < 0.05; Table 4). Compared to HC, BD without GI symptoms were characterized by significantly higher levels of lipid, pantothenate, N-acetyl-glycoprotein, ascorbic acid, valine, 3-D-hydroxybutyric acid, dimethylglycine, and significantly lower levels of acetate, trimethylamine oxide, and guanidine acetate.

**Table 4:** The peak area of metabolites in serum ^1^H-NMR spectra of HC group and non-GI group.

**Metabolites**	**Peak area after normalization**	
	**HC**	**Non-GI**
Lipid	0.345 ± 0.069	0.442 ± 0.075
Pantothenate	0.270 ± 0.032	0.373 ± 0.061
Valine	0.015 ± 0.005	0.097 ± 0.051
3-D-hydroxybutyric acid	1.460 ± 0.385	1.808 ± 0.669
Acetate	0.749 ± 0.069	0.504 ± 0.086
N-acetyl-glycoprotein	0.107 ± 0.025	0.318 ± 0.031
Dimethylglycine	0.079 ± 0.026	0.190 ± 0.036
Trimethylamine oxide	0.813 ± 0.134	0.333 ± 0.047
Guanidine acetate	0.979 ± 0.116	0.516 ± 0.101
Ascorbic acid	0.018 ± 0.015	0.138 ± 0.061

**Figure 6:**
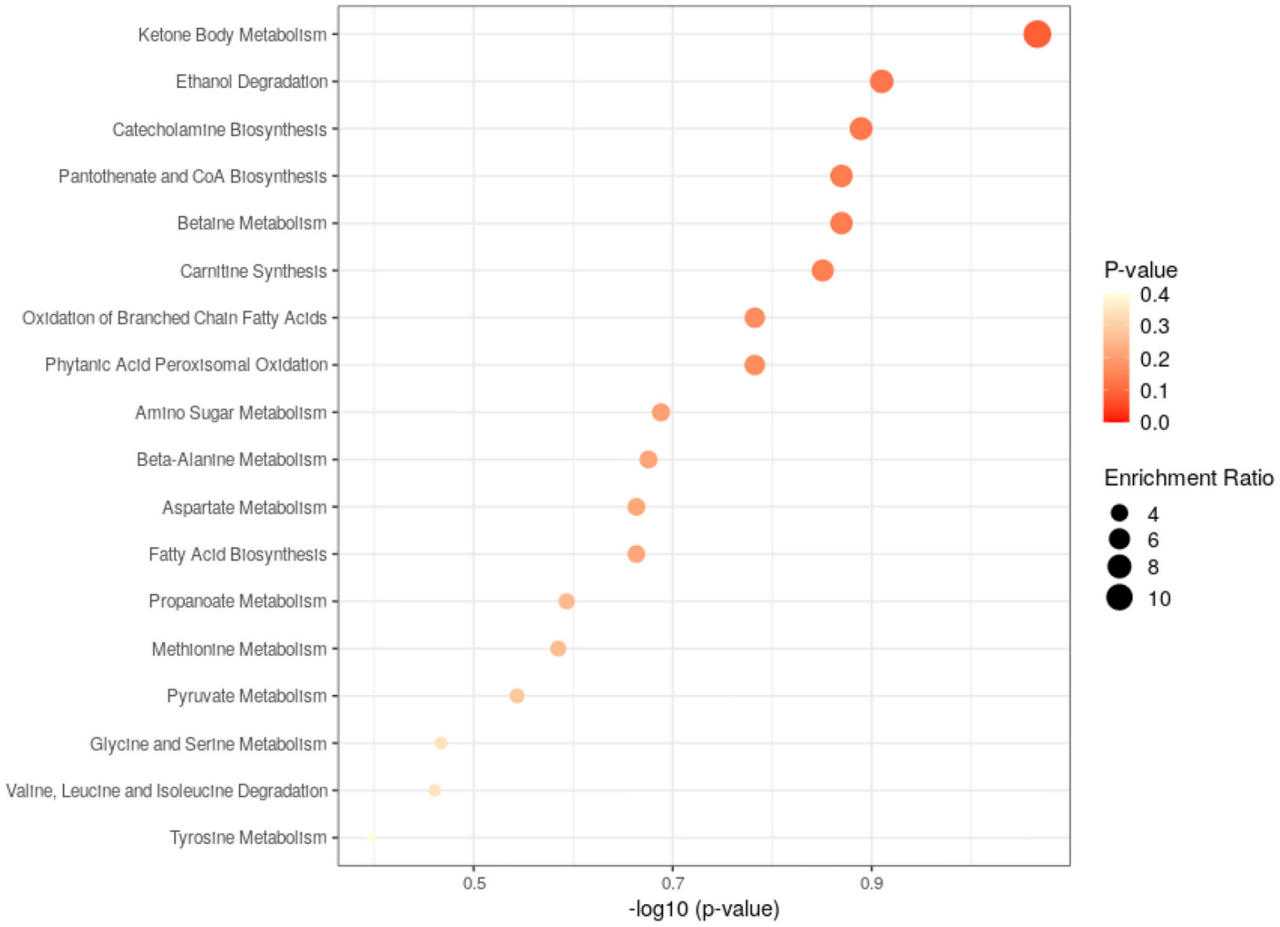
Metabolic pathways analysis of the differential metabolites found between HC and non-GI group.

The closely related metabolic pathways between HC and BD without GI symptoms group was ketone body metabolism. The analysis results are shown in Figure 6.

## Discussion

To the best of our knowledge, this is the first study to investigate the metabolomics characteristics of BD patients with GI symptoms. The results revealed that BD patients with GI symptoms experience more severe symptoms compared to the in metabolic pathways associated with GI symptoms that may be risk factors for gastrointestinal symptoms in BD patients.

In this study, we found that the total HAMD-24 scores in the GI symptoms group were greater than that of the non-GI symptoms group, that consistent with past research findings. Liu et al. demonstrated that the presence of GI symptoms in patients with MDD is associated with more severe symptoms of depression, especially as it pertains to patients who demonstrate no GI symptoms (21). Pinto-Sanchez et al. found a positive correlation between the prevalence and severity of depressive symptoms and the frequency and severity of GI symptoms in patients with FGIDs (22). Perera et al. studied 16,415 patients in the Hispanic Community Health Study and found that adults who reported multiple episodes of GI symptoms were more psychologically stressed than those who reported less frequent episodes of GI symptoms (23). Karling et al. discovered that shown that also patients with an established recurrent depression disorder report high scores on GI symptoms, but when in remission they do not differ from controls in reporting GI symptoms (24), and similarly in BD patients, they also shown that there is a strong association between symptoms of affectivity and GI symptoms (11). We believe that the present study and our previous study support that GI symptoms has an effect on the affective symptoms.

Although the exact pathological alterations in patients with BD are unknown, recent studies have demonstrated extensive alterations in very complex metabolic pathways may partially underlie the pathophysiology, and we found that these changes were independent of dietary habits. Based on metabolomic analysis results, we found that the common disturbances metabolic pathways of both groups of patients jointly exhibit disturbances of ketone body metabolism. In mammals, ketone bodies are produced predominantly in the liver from β-oxidation (FAO)-derived acetyl-CoA, and they are transported to extrahepatic tissues for terminal oxidation. Acetoacetic acid, β-hydroxybutyric acid, and acetone are eventually formed, and these three products are collectively known as ketone bodies. Apart from serving as energy fuels for extrahepatic tissues like brain, heart, or skeletal muscle, ketone bodies play pivotal roles as signaling mediators, drivers of protein post-translational modification (PTM), and modulators of inflammation and oxidative stress (25). Growing preclinical and clinical evidence that dietary ketosis lead to the mitochondrial dysfunction, amelioration of oxidative stress, and peripheral and brain inflammation in animals and humans (13, 26, 27), and research teams have reported that nutritional ketosis may reduce symptoms in patients with schizophrenia (28), BD (29), and major depressive disorder (30, 31). It is means that major depressive disorder, BD, and schizophrenia are increasingly described as neuroprogressive disorders, reflecting progressive neuroanatomical and cognitive decline caused by many common factors present in each disorder, such as peripheral and brain inflammation, oxidative stress, mitochondrial dysfunction with disorders of tryptophan metabolism (32–35). In a word, ketone body metabolism be involved in the inflammation and oxidative stress may be one of the pathogeneses of BD.

What's more, we found the unique disturbances metabolic pathways of BD patients with GI symptoms were fatty acid biosynthesis and tyrosine metabolism. GI disorders pathogenesis involves changes in GI motility, intestinal secretion, visceral hypersensitivity, and intestinal permeability, all of which can be modified by the gut microbiome (36). Recent studies have shown that the composition and metabolic functions of gut microbiota have been proposed as being able to affect fatty acid biosynthesis (37). By comparing the response of germ-free and normal mice to high-fat diets, found that germ-free mice are resistant to high-fat diets, but microbial remodeling can lead to obesity in germ-free mice, thus confirming that gut microbiome are important factors affecting fatty acid biosynthesis (38, 39). Some other studies have shown that the human gut microbiota produces dozens of metabolites that accumulate in the bloodstream, where they can have systemic effects on the host (40). Dodd et al. used a combination of genetics and metabolic profiling to characterize a pathway from the gut symbiont Clostridium sporogenes that generates aromatic amino acid metabolites, and the results reveal that all three aromatic amino acids (tryptophan, phenylalanine and tyrosine) serve as substrates for the pathway, and it involves branching and alternative reductases for specific intermediates, through modulate serum levels of these metabolites in gnotobiotic mice, and show that in turn this affects intestinal permeability and systemic immunity (41). Hence, according previous studies, we found that the abnormalities of these two metabolic pathways may be related to the disturbance of the gut microbiome, and the gut microbiome has been implicated in multiple human chronic GI disorders (17).

## Conclusion

In conclusion, we show that BD patients with GI symptoms have more severe depressive symptoms. BD patients with GI symptoms exhibited abnormalities in fatty acid and tyrosine metabolism, which may be associated with the disturbance of the gut microbiome. Both two patient groups exhibit abnormalities in the ketone body metabolism, which may serve as a biomarker for the pathogenesis of BD patients.

### Limitations

There are two primary methodological limitation for the current study that should be considered. First, our study is a cross-sectional study that we could not continue to explore the changes in metabolomics with drug treatment. Secondly, the sample size of this study was not large. Therefore, we plan to conduct further large sample follow-up studies.

## Data Availability Statement

The raw data supporting the conclusions of this article will be made available by the authors, without undue reservation.

## Ethics Statement

The studies involving human participants were reviewed and approved by Shanxi Bethune Hospital. The patients/participants provided their written informed consent to participate in this study.

## Author Contributions

Y-BX and YR contributed to manuscript preparation. Y-BX, J-ST, and W-ZW performed the data analysis and statistics. YJ contributed in oversaw data/demographic data collection. X-JG, Y-BX, and YJ wrote and revised the manuscript. HY and YR was in charge of design, implementation of the study, and contributed to data interpretation. All authors contributed to the article and approved the submitted version.

## Funding

This work was supported by the National Natural Science Foundation of China (8210053813), Research Project Supported by Shanxi Scholarship Council of China (2021-167), Applied Basic Research Projects of Shanxi Province China (201901D111418), and Scientific Research Project of Health Commission of Shanxi Province (2019007).

## Conflict of Interest

The authors declare that the research was conducted in the absence of any commercial or financial relationships that could be construed as a potential conflict of interest.

## Publisher's Note

All claims expressed in this article are solely those of the authors and do not necessarily represent those of their affiliated organizations, or those of the publisher, the editors and the reviewers. Any product that may be evaluated in this article, or claim that may be made by its manufacturer, is not guaranteed or endorsed by the publisher.
